# Human amniotic epithelial cells differentiate into cells expressing germ cell specific markers when cultured in medium containing serum substitute supplement

**DOI:** 10.1186/1477-7827-10-108

**Published:** 2012-12-14

**Authors:** Ayelet Evron, Shlomit Goldman, Eliezer Shalev

**Affiliations:** 1 Laboratory for Research in Reproductive Sciences, Department of Obstetrics and Gynecology, Emek Medical Center, Afula, Israel; 2 Rappaport, Faculty of Medicine, Technion, Haifa, Israel

**Keywords:** Amniotic epithelial cells, Germ cell specific markers, Differentiation

## Abstract

**Background:**

Human amniotic epithelial cells (hAECs) maintain the plasticity of pregastrulation embryonic cells, having the potential to differentiate into all three germ layers. The potential of these cells to differentiate into cells expressing germ cell specific markers has never been described before.

**Methods:**

In the present study, hAECs were cultured in medium containing serum substitute supplement (SSS). Gene and protein expression of germ cell and oocyte specific markers was assessed by reverse transcription-polymerase chain reaction **(**RT-PCR), immunofluorescence staining and flow activated cell sorter analysis (FACS) in hAECs at different time points during the differentiation into cells expressing germ cell specific markers.

**Results:**

When cultured with SSS, already at passage 1, hAECs start to express the germ cell specific genes *C-KIT, DAZL, VASA* and *ZP3* and at passage 5 large round cells, resembling oocytes, appeared. The cells express the germ cell specific marker DAZL, the oocyte specific markers GDF9 and ZP3 and the meiosis specific markers DMC1 and SCP3 at the protein level.

**Conclusions:**

From our preliminary results we can conclude that hAECs have the potential to differentiate into cells expressing germ cell specific markers.

## Background

Human amniotic epithelial cells (hAECs) develop from the epiblast. The differentiation occurs within 8 days after fertilization, before gastrulation and specification of the three germ layers. For this reason the hAECs maintain the plasticity of pregastrulation embryo cells [1]. It has been shown that hAECs have the potential to differentiate in vitro to ectoderm cells (neural cells) [1,2], mesodermal cells (cardiomyocytes) [1,2] and endodermal cells (hepatocytes and pancreatic cells) [1-3].

Germ cells are the biological route for genetic transmission from one generation to the next. These cells constitute a very different cell population from somatic cells, with unique characteristics, and display a haploid chromosomal number after a delicate process of meiosis [4].

Germ cell development has been difficult to study in vivo, because important early events occur after implantation. This difficulty is most evident in humans, when ethical issues are considered. In addition, limitations in the number of germ cells, their deep location within the embryo and the fact that germ cells migrate during development mitigates the degree to which gametogenesis can be effectively studied in vivo [5]. For that reason, a human stem cell based in vitro system could provide a valuable platform to study some aspects of germ cell development.

The formation of germ cells from the epiblast in the embryo during gastrulation involves segregation of the primordial germ cells (PGCs) from the somatic lineages and migration of PGCs to the genital ridges. During the colonization of the genital ridge further maturation of the gonocytes involves proliferation and apoptosis before they differentiate into mature gametes within the gonads in response to external paracrine signals [5]. C-KIT, a tyrosine kinase receptor is expressed during the migration of PGCs into the genital ridge [4]. DAZL, an RNA binding protein, and VASA, an ATP dependent RNA helicase, are expressed in post migratory germ cells during the colonization of the genital ridge [6]. Oocyte specific markers are ZP3 and growth differentiation factor 9 (GDF9). ZP3 is a glycoprotein of the zona pellucida [7] and GDF9, a member of the transforming growth factor beta (TGF beta) superfamily, is a factor secreted specifically by oocytes and involved in folliculogenesis [8].

The ability of germ cell like cells derived from embryonic stem cells to undergo meiosis was investigated by several groups [9,10]. The expression of *STRA8* (stimulated by retinoic acid gene 8) is known to play an important role in the initiation of meiosis in germ cells [11]. DMC1 (dosage suppressor of mck 1 homolog) is a meiosis specific RECA/RAD51 homolog required for recombination repair of meiotic DNA double stranded breaks and SCP3 (synaptonemal complex protein 3) is regarded as a marker for identifying meiotic transition as its expression is specific to meiosis and it is present from the initiation of meiosis [11].

Due to the ease in obtaining hAECs from an ethical uncontroversial and abundant source, the human placenta, and their proved pluripotency, hAECs may bear the potential to provide an appropriate in vitro system for the investigation of some aspects of human gametogenesis.

The aim of the current study was to explore the possibility that hAECs can be directed to differentiate into cells expressing germ cell specific markers.

## Methods

### Tissue collection

Placentas were obtained after uncomplicated vaginal deliveries or cesarean sections from healthy mothers with written informed consent. For the tissue collection, ethical approval was obtained from the ethical committee at the Emek Medical Center, Afula, Israel. The amnion layer was mechanically peeled off of the chorion and transported in PBS, supplemented with antibiotics, to the laboratory. In the laboratory the amnion was washed several times with PBS, supplemented with antibiotics, to remove blood.

### Isolation of hAECs

To release hAECs, first the amnion membrane was placed in a 50ml centrifugation tube (BD Falcon, Franklin Lakes, NJ, USA) containing 10ml 0.25% Trypsin/EDTA (Kibbutz Beit-Ha’Emek, Israel) and shaken at room temperature for 30 seconds. Then, the amnion membrane was transferred into two new 50ml centrifugation tubes (Falcon), each containing 15ml 0.25% Trypsin EDTA (Beit-Ha’Emek) and shaken in a Comfort shaker (Comfort. Heto Master Shake, Heto-Holten A/S Type: SBD50-1, Paris, France) at 200 rpm (12 × g) at 37°C for 10 minutes. The cells from the first 10 minutes of digestion were discarded to exclude debris. The amnion membrane was then transferred into two new 50ml centrifugation tubes (Falcon), each containing 15 ml 0.25% Trypsin/EDTA (Beit-Ha’Emek) and shaken in a Comfort shaker (Comfort. Heto Master Shake) at 37°C for 30 minutes. A second 30 minute incubation was performed and at the end of the process the amnion membrane was discarded. To the first and second 30 minute digests 10ml of standard medium was added and the digests were centrifuged at 1300 rpm in order to remove trypsin. Cells were pooled, filtered through a 100μm cell strainer and counted in a hemocytometer [12].

### Cell culture

hAECs were plated on 100mm or 60mm diameter plastic petri dishes (Falcon) at a density of 12.7×10^4^ cells per cm^2^ in medium containing SSS to induce differentiation into cells expressing germ cell specific markers or in standard culture medium as a control. Standard culture medium is Dulbecco's modified Eagle's medium (Beit-Ha’Emek) supplemented with 20% fetal calf serum, 2mM L-glutamine, 1% nonessential amino acids, 1mM sodium pyruvate, 1% antibiotic-antimycotic (all from Beit-Ha’Emek), 55μM 2-mercaptoethanol (Sigma-Aldrich, St. Louis, MO, USA) and 10ng/ml epidermal growth factor (Sigma-Aldrich). In medium containing SSS, fetal calf serum was replaced by 20% SSS (Irvine Scientific, Santa Ana, CA). SSS (Irvine Scientific) consists of 6% total protein (weight/volume) in normal saline. The protein component contains 84% human serum albumin from a therapeutic grade source material and 16% alpha and beta globulins.

Cell cultures were maintained in a humidified atmosphere containing 5% CO_2_ at 37°C and medium was replaced every three to four days. Cells were passaged every 8 days by harvesting them from tissue culture dishes by 0.25% Trypsin/EDTA (Beit-Ha’Emek), centrifuging and re-culturing.

### Immunofluorescence staining

For immunofluorescence analysis, freshly isolated hAECs and hAECs cultured in medium containing SSS or in standard medium from passage 1, passage 3 and passage 5 were seeded on cover glasses (Menzel GmbH, Braunschweig, Germany) at a density of 12.7×10^4^ cells per cm^2^ and cultured in medium containing SSS or in standard medium. After 24 hours cultured cells were washed 3 times with PBS and fixed with 4% paraformaldehyde (Electron Microscope Sciences, Belgar) in PBS for 10 minutes at 4°C, then washed twice with PBS and permeabilized for 5 minutes at 4°C with 0.2% Triton (Sigma-Aldrich) in PBS. After a PBS wash, cells were incubated for 30 minutes with blocking buffer (PBS supplemented with 3% BSA), then washed two times with PBS and incubated for 30 minutes at room temperature with primary antibodies (goat polyclonal anti-human DAZL sc-27333, goat polyclonal anti-human GDF9 sc-12244, rabbit polyclonal anti-human ZP3 sc-25802, rabbit polyclonal anti-human SCP3 sc-33195 and rabbit polyclonal anti-human DMC1 sc-22768; all from Santa Cruz Biotechnology, Santa Cruz, CA, USA) 1 μg per cover glass in 700 μl PBS supplemented with 1.5% BSA. After five washings with PBS, cells were incubated for 30 minutes in the dark at room temperature with secondary fluorescein-labeled antibodies; for DAZL and GDF9: donkey anti-goat IgG-FITC sc-2024 (Santa Cruz Biotechnology) and for ZP3, SCP3 and DMC1: goat anti-rabbit IgG conjugated with AlexaFluor-546 (Invitrogen Molecular Probes, Eugene, Oregon, USA), 0.5 μg per cover glass in 700 μl PBS supplemented with 1.5% BSA. Following two washings with PBS, cells were incubated with 1μg/ml DAPI (GX12369, Inno-Train Diagnostik GmbH, Kronberg/Taunus, Germany) for 1–2 minutes at room temperature in the dark, washed two times with PBS and mounted with DPX (Sigma-Aldrich) onto microscope slides (Superfrost, Menzel GmbH). Negative controls were run in parallel by omitting the primary antibody or replacing the primary antibody with goat and respectively rabbit IgG isotype control antibody (Santa Cruz Biotech) at the same concentration as the primary antibody (1 μg pr slide in 700 μl PBS supplemented with 1.5% BSA). No specific immuno-reactivity was detected in these negative control specimens. Stained cells were photographed at a magnification of ×63 or ×100 using an upright fluorescence microscope Axioscop 2 (Carl Zeiss GmbH, Hamburg, Germany) and a CCD camera hooked to the system (Roper Scientific Camera, Roper Industries Inc., Sarasota, Florida, USA). Exposure time and gain offset were maintained between the images from each experiment.

### FACS

For FACS, GDF9 protein expression was evaluated in hAECs cultured in medium containing SSS at passage 5. The primary antibody to detect the expression of the oocyte specific marker was goat polyclonal anti-human GDF9 sc-12244 (2μg/ml) (Santa Cruz Biotechnology). Secondary fluorescein-labeled antibody for GDF9 was donkey anti-goat IgG-FITC sc-2024 (Santa Cruz Biotechnology). Negative controls were run in parallel by omitting the primary antibody or by replacing the primary antibody with goat IgG isotype control antibody (Santa Cruz Biotech) at the same concentration as the primary antibody. Cells (1×10^6^ cells/ml) were washed in PBS (Beit-Ha’Emek) containing 5% fetal calf serum (Beit-Ha’Emek) at 1250 rpm, 4°C for 10 minutes and fixed with 4% paraformaldehyde (Electron Microscope Sciences, Belgar) at 37 °C for 10 minutes. After chilling in ice for 1 minute cells were permeabilized by incubating cells for 30 minutes on ice with ice cold 90% methanol. After washing in PBS containing 5% fetal calf serum at 1250 rpm, 4°C for 10 minutes, cells were incubated with the primary antibody for 45 minutes at room temperature. After three washings in PBS containing 5% fetal calf serum at 1250 rpm, 4°C for 10 minutes each time, cells were incubated with the secondary, fluorescein-conjugated antibody at room temperature for 30 minutes in the dark. After three washings in PBS containing 5% fetal calf serum at 1250 rpm, 4°C for 10 minutes each time, 500μl PBS containing 1% paraformaldehyde (Electron Microscope Sciences, Belgar) was added to the pellet and the cell solution was stored at 4°C in the dark until analysis. Cells were analyzed on a flow cytometer (FACS Calibur, CA, USA). Five to ten thousand events were acquired per sample with fluorescence measured on logarithmic scales. Forward and side light scatter gates were set to exclude dead cells, debris, and clumps of cells. Autofluorescence was removed from the samples by setting gates on unstained controls.

### RNA isolation for RT-PCR

For RT-PCR, RNA was isolated from freshly isolated hAECs and from hAECs cultured in medium containing SSS or in standard medium at the end of each passage using the EZ-RNA Total RNA isolation kit (Beit-Ha’Emek) according manufacturer's instructions. RNA concentration was determined spectrophotometrically. The assessment of a 260/280 optical density (O.D.) ratio of 1.6 to 1.9 confirmed that the final preparation of total RNA was free of DNA and proteins.

### First strand cDNA synthesis for RT-PCR

To obtain the cDNA, 5 μg RNA was denatured at 70°C for 10 min and then reverse transcribed in the presence of 25 ng/μl random primer (Promega, Mannheim, Germany), 2.5 mM MgCl_2_, 0.5 mM deoxy-NTPs, 10 mM dithiothreitol, and 10 U ribonuclease H- reverse transcriptase (SuperScript II RT, Life Technologies, Inc Invitrogen, Dorset, UK.) for 60 min at 42°C, and 5 min at 95°C. Subsequently, 3 μl of the resulting cDNA was used as a template for PCR.

### RT-PCR

The 1^st^ strand cDNA product, 3 μl, was subsequently amplified in a total volume of 30 μl PCR assay, containing 6 μl Red load Taq master (LAROVA GmbH, Teltow, Germany), 0.5 μM of each primer and filled up to a total volume of 30 μl with sterile PCR grade water. Primers for Glyceraldehyde 3-phosphate dehydrogenase (*GAPDH)*, *C-KIT*, *DAZL*, *VASA*, *ZP3*, *DMC1*, *SCP3* and *STRA8* were used.

The PCR amplification for all primers was performed for 30 cycles in an automated thermocycler profile. Before starting each primer was normalized for sub-saturation condition. We used a series of dilutions of a bulk RNA preparation to determine sub-saturation conditions for the PCR products using *GAPDH*. The amplification parameters were denaturation at 94°C for 5 minutes, followed by 30 cycles as follows: denaturation at 94°C for 30 seconds, annealing at 57°C for 1 minute (for *GAPDH*, *C-KIT*, *DAZL*, *VASA* and *ZP3* primers), or respectively annealing at 56°C for 1 minute (for *DMC1*, *SCP3* and *STRA8* primers) and elongation at 72°C for 90 seconds and at the end of the program 72°C extension for 10 minutes. The sequences of appropriate primers were obtained from the gene bank and prepared at IDT Inc., Hy-Labs, Rehovot, Israel. Product sizes, annealing temperatures and primer sequences are listed in Table 1. RT-PCR products were analyzed by 2.5% agarose ethidium bromide gel electrophoresis. Images were captured with Polaroid film (Hertfordshire, UK) under UV light. Amplification of the housekeeping gene *GAPDH* transcripts was performed simultaneously to confirm RNA integrity, efficiency and for quantification of cDNA. Negative control reactions containing samples without cDNA or Taq enzyme were used.

**Table 1:** Primer sequences and the conditions of RT-PCR

**Primer**	**Sequence (5’ → 3’)**	**Product size (bp)**	**Annealing temperature (°C)**	**Cycles**
*GAPDH*	FW- TGATGACATCAAGAAGGTGGTGAAG	230	57	30
	REV -TCCTTGGAGGCCATGTGGGCCAT			
*C-KIT*	FW- TTGTGCACGACGATGTCTGA	296	57	30
	REV- AGTCCATACCTCCCTCTCTT			
*DAZL*	FW- GAGTGGTCAAAGGAGCCAAA	144	57	30
	REV- AGGAGCCACCTCCCTGAG			
*VASA*	FW- TCTTCACAAGCTCCCAATCC	165	57	30
	REV- TGAGAATACAAGGACAGGAGCT			
*ZP3*	FW- GCTTTGCCTTCTGACACCTC	127	57	30
	REV- CAGACACAGGGTGGGAGG			
*DMC1*	FW- AGCAGCAAAGTTCCATGAAG	300	56	30
	REV- TGAGCTCTCCTCTTCCCTTT			
*SCP3*	FWD-TGGAAAACACAACAAGATCA	344	56	30
	REV-GCTATCTCTTGCTGCTGAGT			
*STRA8*	FW-CCTCAAAGTGGCAGGTTCTGAA	126	56	30
	REV-TCCTCTAAGCTGCTTGCATGC			

### Statistical analysis

For FACS analysis results were expressed as mean ± SD of six independent experiments.

## Results

### Morphology of hAECs cultured in medium containing SSS

**Figure 1:**
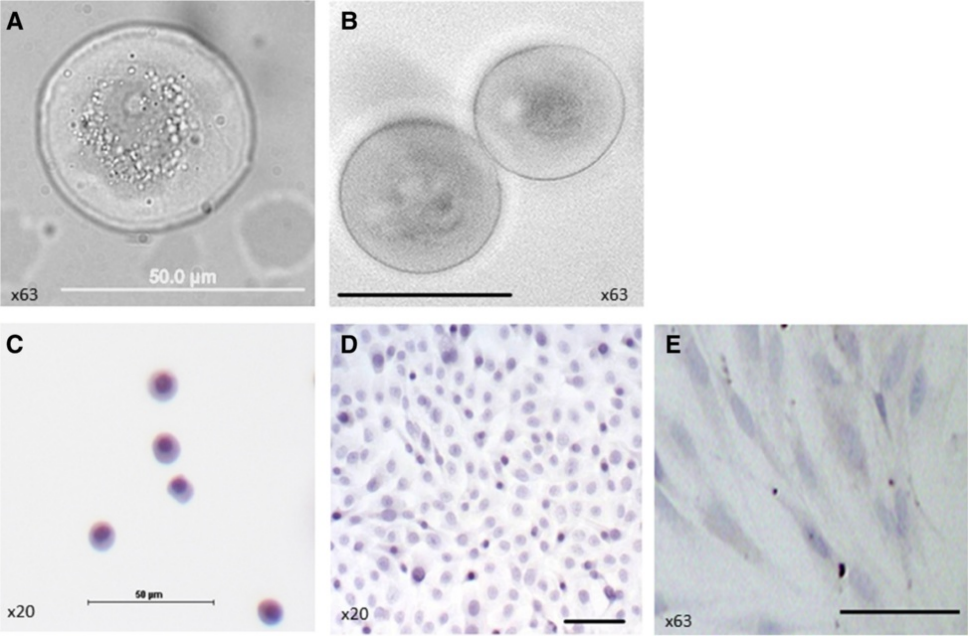
**Morphology of hAECs cultured in medium containing serum substitute supplement (SSS).** Bright field micrograph of (**A**) loosely attached cell at passage 5; (**B**) floating cells at passage 5. Hematoxylin staining of (**C**) Freshly isolated hAECs; (**D**) hAECs at passage 1; (**E**) hAECs cultured in standard medium containing fetal calf serum at passage 5.

Large round loosely attached cells (Figure 1A) and large round floating cells (Figure 1B) with a diameter of about 50μm were detected in hAECs culture grown in medium containing SSS at passage 5. These large round cells are much bigger in size compared to freshly isolated hAECs (Figure 1C) and possess different morphology compared to hAECs cultured in medium containing SSS at passage 1 (Figure 1D). No such large round cells were observed in hAECs cultured in standard medium containing fetal calf serum (Figure 1E).

### Protein expression of germ cell and oocyte specific markers

**Figure 2:**
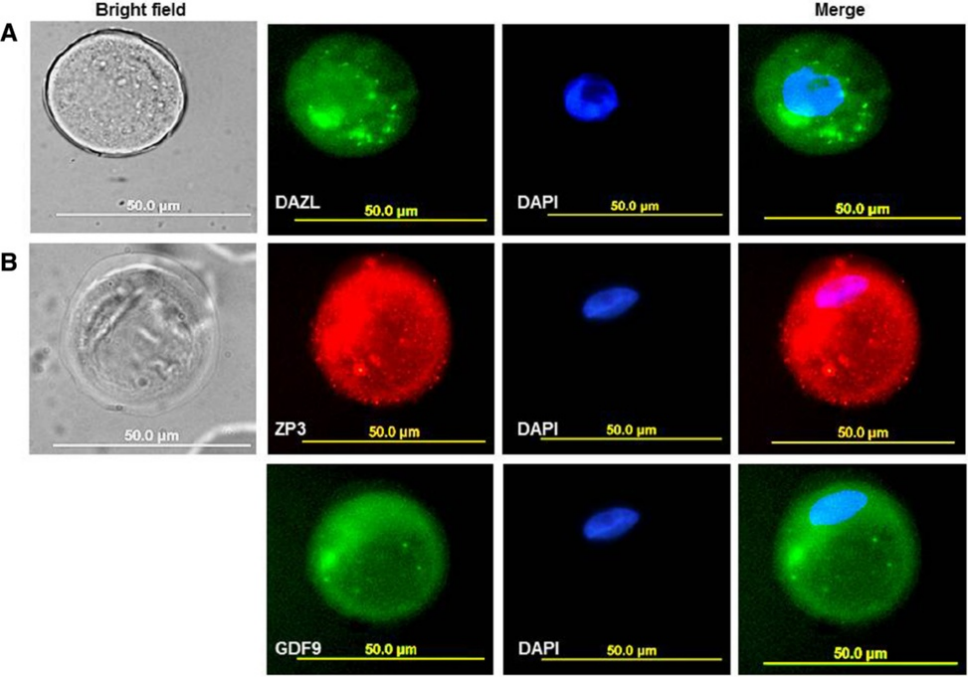
**Immunofluorescence staining of hAECs cultured in medium containing SSS at passage 5.** Large round cells, which expresses (**A**) DAZL (green); (**B**) ZP3 (red) and GDF9 (green). On the left side are bright field micrograph of the same cells; Blue color, DAPI nuclear staining, magnification in all x63.

**Figure 3:**
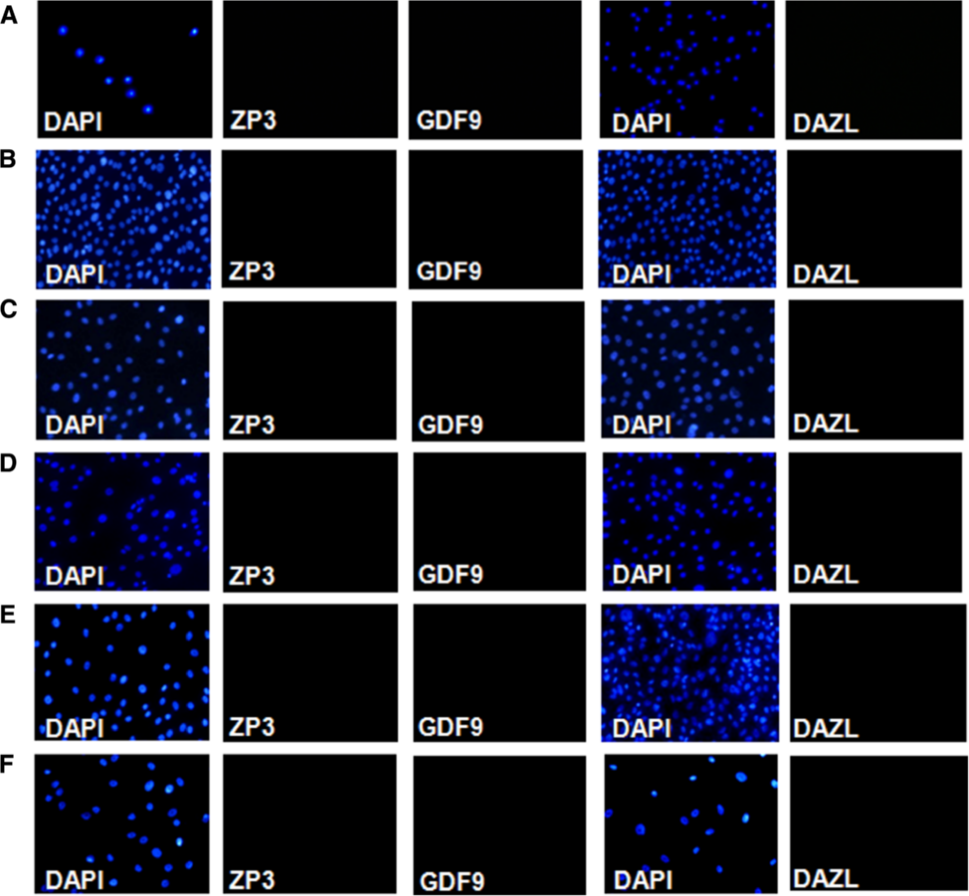
**Immunofluorescence staining of hAECs negative for DAZL, ZP3 and GDF9.** (**A**) Freshly isolated hAECs; (**B**) hAECs cultured in medium containing SSS at passage 1 and at passage 3 (**C**). hAECs cultured in standard medium at passage 1 (**D**), at passage 3 (**E**) and at passage 5 (**F**). Blue color, DAPI nuclear staining of the corresponding cells.

For immunofluorescence analysis, freshly isolated hAECs and hAECs cultured in medium containing SSS or in standard medium from passage 1, passage 3 and passage 5 were seeded on cover glasses at a density of 12.7×10^4^ cells per cm^2^ and cultured in medium containing SSS or in standard medium. After 24 hours culture expression of germ cell and oocyte specific markers was assessed by immunofluorescence staining. Representative pictures of the results from five independent experiments are shown in Figure 2 and Figure 3. Expression of DAZL, GDF9 and ZP3 was observed in large round cells in hAECs culture at passage 5 grown in medium containing SSS (Figure 2). In contrary no expression of DAZL, GDF9 and ZP3 was observed in freshly isolated hAECs (Figure 3A), in hAECs culture at passage 1 and at passage 3 grown in medium containing SSS (Figure 3B and C) and in hAECs culture at passage 1,3 and 5 grown in standard medium (Figure 3D, E and F).

**Figure 4:**
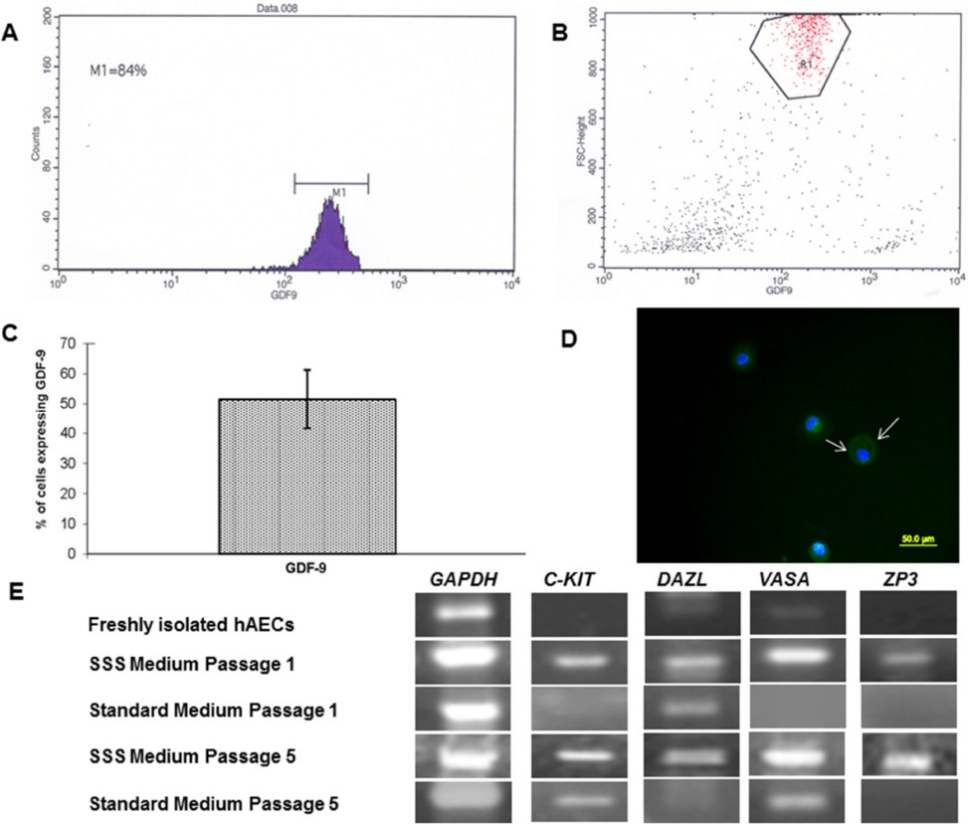
**FACS analysis and RT-PCR of germ cell specific markers.** (**A-C**) FACS analysis (**A**) representative histogram, (**B**) dot plot of one of the six independent experiments (**C**) bar graph describes mean ± SD of six independent experiments. (**D**) Immunofluorescence staining of GDF9 in a group of hAECs cultured in medium containing SSS at passage 5 (magnification x20); white arrows, hAECs stained for GDF9; Green color, GDF9; Blue color, DAPI nuclear staining. (**E**) RT-PCR of *C-KIT*, *DAZL*, *VASA* and *ZP3* gene expression in freshly isolated hAECs and in hAECs cultured in medium containing SSS and respectively in standard medium at passage 1 and 5, *GAPDH* gene expression was used as an internal control. Shown are photographs of the representative bands of the gene products in a 2.5% agarose gel.

FACS analysis of six independent experiments demonstrated that 51.48% ± 19.6% of cells in hAECs culture grown in medium containing SSS at passage 5 express GDF9 at the protein level (Figure 4A, B and C). Immunofluorescence staining of GDF9 in hAECs cultured in medium containing SSS at passage 5 confirms that about 50% of the cells express GDF9 (Figure 4D).

#### Gene expression of germ cell specific markers

RT-PCR was performed in order to assess the absence or presence of mRNA in an “all or nothing” approach. For this, hAECs were plated on 100mm or 60mm diameter plastic petri dishes at a density of 12.7×10^4^ cells per cm^2^ in medium containing SSS to induce differentiation into cells expressing germ cell specific markers or in standard medium as a control. After each passage or immediately after the isolation of hAECs from amniotic membranes RNA was extracted, reverse transcribed and RT-PCR products were analyzed by 2.5% agarose ethidium bromide gel electrophoresis. Photographs of representative bands of gene products from five independent experiments are shown in Figure 4E. In freshly isolated hAECs no gene expression of *C-KIT* and *ZP3* was detected, while the expression of *DAZL* and *VASA* was very weak (Figure 4E). In hAECs cultured in medium containing SSS already at passage 1 gene expression of *C-KIT*, *DAZL*, *VASA* and *ZP3* appeared and was still visible at passage 5 (Figure 4E). On the other hand, in hAECs cultured in standard medium containing fetal calf serum no gene expression of *C-KIT*, *VASA* and *ZP3* was observed at passage 1, but expression of *DAZL* was detected. At passage 5 in hAECs cultured in standard medium expression of *C-KIT* and *VASA* appeared, however *DAZL* gene expression disappeared and no expression of *ZP3* was observed (Figure 4E).

### Gene and protein expression of meiosis specific markers

**Figure 5:**
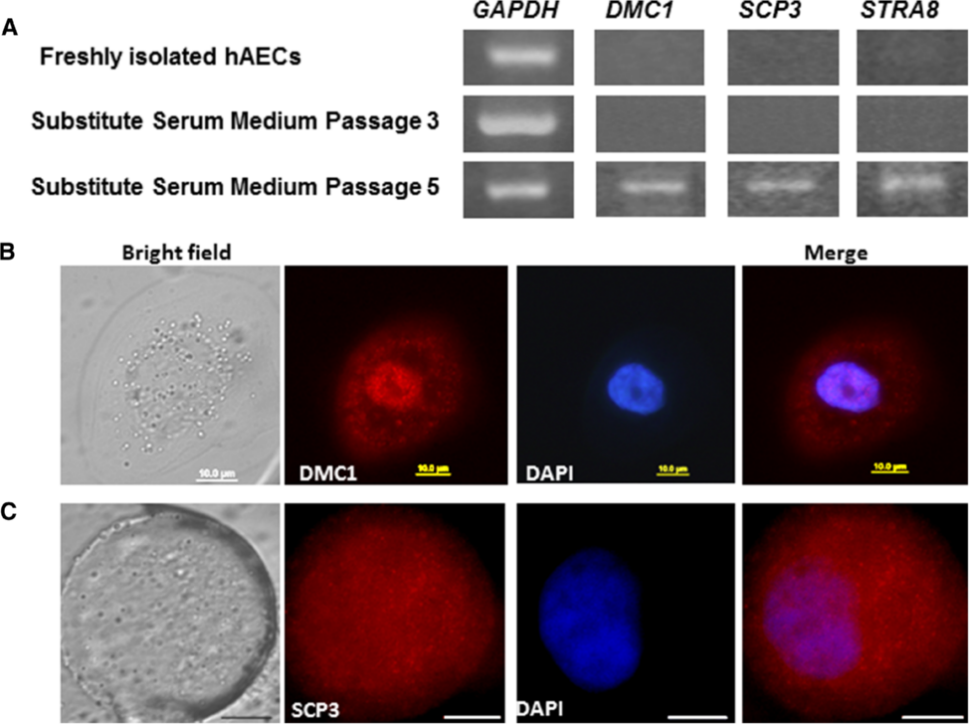
**Gene and protein expression of meiosis specific markers.** RT-PCR of *DMC1*, *SCP3* and *STRA8* gene expression in freshly isolated hAECs and in hAECs cultured in medium containing SSS at passage 3 and passage 5. Photographs of representative bands of the gene products in a 2.5% agarose gel from four independent experiments are shown. *GAPDH* gene expression was used as an internal control. (**B** and **C**) Immunofluorescence staining of hAECs grown in medium containing SSS at passage 5, (**B**) nuclear and cytoplasmic DMC1 expression (red); (**C**) cytoplasmic SCP3 (red). On the left side are bright field micrograph of the same cells, scale bar = 10μm; magnification x100. Blue color, DAPI nuclear staining.

RT-PCR was performed in order to assess the absence or presence of mRNA in hAECs in an “all or nothing” approach. Gene expression of the meiosis specific markers *DMC1*, *SCP3* and *STRA8* was investigated in freshly isolated hAECs and in hAECs cultured in medium containing SSS at passage 3 and passage 5. Gene products were analyzed by 2.5% agarose ethidium bromide gel electrophoresis. Photographs of representative bands of gene products from four independent experiments are shown in Figure 5A. Gene expression of *DMC1*, *SCP3* and *STRA8* was detected in hAECs cultured in medium containing SSS at passage 5, while none of the investigated meiosis specific genes were expressed in freshly isolated hAECs and in hAECs cultured in medium containing SSS at passage 3 (Figure 5A).

Protein expression of the two meiosis specific markers DMC1 and SCP3 in hAECs cultured in medium containing SSS at passage 5 was assessed by immunofluorescence staining. Representative pictures of the results from eight independent experiments are shown in Figure 5B and C. The large round cells express both meiosis specific markers at the protein level (Figure 5B and C). While SCP3 expression was detected only in the cytoplasm (Figure 5C), DMC1 was found to be expressed in the cytoplasm and diffusely expressed in the nucleus with no clear staining pattern characteristic for any of the stages (leptotene, zygotene, pachytene and diplotene) during prophase of the first meiotic division (Figure 5B).

## Discussion

Previous studies have shown that germ cell like cells can be generated from embryonic stem cells [13] and somatic stem cells [7,14]. In the present study, we show for the first time the generation of cells expressing germ cell specific markers from hAECs. We observed in hAECs culture grown in medium containing SSS at passage 5 large round floating and large round loosely attached cells with a diameter of about 50μm. Gene expression analysis revealed that these large round cells express germ cell and oocyte specific markers. No expression of *C-KIT* and *ZP3* was observed in freshly isolated hAECs. However, freshly isolated hAECs expressed very low levels of the genes *DAZL* and *VASA*. This suggests that hAECs possess the potential to differentiate into cells expressing germ cell specific markers. Amniotic epithelial cells develop from the epiblast by 8 days after fertilization and before gastrulation [1] and PGCs develop from the proximal epiblast during gastrulation [5]. Due to the derivation of the amniotic epithelium before the time of gastrulation, the amniotic epithelium may contain cells with the intrinsic potential to develop into cells expressing germ cell specific markers.

Immunofluorescence staining revealed that the large round cells observed in hAECs culture grown in medium containing SSS at passage 5 express the germ cell specific marker DAZL and the oocyte specific markers ZP3 and GDF9 at the protein level. About 51% of hAECs cultured in medium containing SSS expressed GDF9 at passage 5. This may be due to the fact that medium containing SSS supports the survival of hAECs differentiating into cells expressing germ cell specific markers more compared to other cell types in the heterogeneous hAECs culture. Thus, the relatively high percentage of GDF9 expressing cells may be due to an enrichment effect of cells undergoing differentiation into germ cell specific marker expressing cells in a heterogeneous hAECs population during culturing and passaging of hAECs in medium containing SSS.

We have demonstrated that hAECs differentiate into cells expressing germ cell specific markers when cultured in medium containing SSS after the cells underwent five to six times trypsinization and re-seeding. SSS is a serum substitute used in assisted reproductive procedures as protein supplements for culture media for gamete and embryo manipulation [15]. SSS contains human serum albumin from a therapeutic grade source material and alpha and beta globulins. The replacement of fetal calf serum with SSS removes several growth factors, amino acids, hormones, trace elements and extracellular matrix components, all which may induce spontaneous differentiation of hAECs into several different cell types and thus decrease the efficiency to give rise to germ cell specific marker expressing cells. Albumin balances the osmolality and scavenges potentially harmful molecules and metal ions that can act as a source of free oxygen radicals [16]. Furthermore, it has been shown that media containing serum albumin as a serum substitute promoted in vitro follicular growth and oocyte maturation more compared to media containing fetal calf serum [17]. The medium in which hAECs differentiated into cells expressing germ cell specific markers also contain epidermal growth factor (EGF), beta-mercaptoethanol, pyruvate, glutamine and nonessential amino acids. Beta-mercaptoethanol is a low molecular weight thiol and supplementation of beta-mercaptoethanol in the *in vitro maturation* (IVM) medium has shown to increase intracellular glutathione content in bovine oocytes and to improve embryo development and quality in several species [18]. It has been suggested that glutathione protects cells from oxidative damage [19]. Thus, increased intracellular glutathione induced by beta-mercaptoethanol may provide cells with a better intracellular condition, preventing oxidative damage and possibly promoting induced germ cell differentiation and oocyte development. Supplementation of medium with EGF may be of benefit to induce the differentiation of hAECs into germ cell specific marker expressing cells due to its positive role in various other mammalian IVM and embryo culture systems [20]. Basic research has shown that glucose is a common energy source for almost all types of animal cells. However, over the last few decades, it has become clear that mature oocytes of many mammalian species preferentially metabolize pyruvate and have little capacity for the metabolism of glucose [21]. Culture medium supplemented with pyruvate may deliver an ideal energy source for cells undergoing differentiation into germ cells and thus support the derivation of germ cell specific marker expressing cells from hAECs.

The large round cells observed in hAECs culture grown in medium containing SSS at passage 5 express meiosis specific markers. Meiosis is a process resulting in the formation of haploid gametes [22]. Prophase of the first meiotic division is subdivided into leptotene, zygotene, pachytene and diplotene stages [23], during which recombination of the homologue chromosome pairs occurs. DMC1, a meiosis specific recombinase [24] is involved during homologous chromosome recombination [25]. For this, a meiosis-specific structure, the synaptonemal complex is formed, which is composed of meiosis specific proteins including SCP3 [22]. Each of the two meiosis specific proteins, SCP3 and DMC1, shows a characteristic protein expression pattern during prophase of the first meiotic division which is distinctive to each stage of prophase I [11]. Thus, protein expression patterns of SCP3 and DMC1 have been widely used in order to characterize meiotic progression of stem cell derived germ cell like cells [9,11]. In the present study, the staining patterns of DMC1 and SCP3 observed in the large round cells obtained from hAECs cultures in medium containing SSS at passage 5 were not characteristic for any stage during prophase of the first meiotic division. The nuclear staining of DMC1 in the large round cells does not match any typical staining pattern during prophase I. In addition, SCP3 is expressed in the cytoplasm of the large round cells.

The failure to propagate meiosis may be due to errors happening during the differentiation process of hAECs into cells expressing germ cell specific markers, such as disturbances in the establishment of specific epigenetic imprints during the early differentiation process, or due to faults occurring during the later stages of differentiation of the large round cells cultured in medium containing SSS at passage 5.

Recently published studies report the derivation of haploid germ cells from human induced pluripotent stem cells [26] and human embryonic stem cells [27]. Despite the fast progress in this specific field of reproductive sciences, yet in most of the studies that reported the derivation of stem cell derived oocyte like cells, the formation of haploid oocytes is lacking due to failure of the oocyte like cells to propagate properly through prophase of the first meiotic division [9,11,13]. Thus, one of the problems encountered in stem cell derived germ cell like cells, seems to be the intact meiotic propagation.

## Conclusions

In the present study hAECs were found to have the potential to differentiate into diploid cells expressing germ cell specific markers. With an eye to the future, possible derivation of similar but haploid cells will offer an appropriate in vitro system for the study of human germ cell specification and gametogenesis.

## Competing interests

The authors declare that they have no competing interest.

## Author’s contributions

A. Evron carried out the laboratory work, participated in design of the study, performed the statistical analysis and drafted the manuscript. S. Goldman participated in conceiving and designing of the study, directed the laboratory work, helped in the statistical analysis and in drafting of the manuscript. E. Shalev conceived and designed the study analyzed the results and edited the manuscript. All authors read and approved the final manuscript.
